# Supplementary material for: Correlation of ammonia and blood laboratory parameters with hepatic encephalopathy: A systematic review and meta-analysis
Source: PLoS One. 2024 Sep 3;19(9):e0307899. doi: 10.1371/journal.pone.0307899 (PMC11371226; doi:10.1371/journal.pone.0307899)
Supplement: S1 File — (DOCX) [file pone.0307899.s001.docx]

**Supplementary file**

**Correlation of Ammonia and** **Blood Laboratory Parameters with Hepatic Encephalopathy: A Systematic Review and Meta-Analysis**

**Ali Sepehrinezhad^1,2^. Negin Ghiyasi Moghaddam^1,†^. Navidreza Shayan^1,†^. Sajad Sahab Negah^1,2,3,*^**

^1^ Neuroscience Research Center, Mashhad University of Medical Sciences, Mashhad, Iran

^2^ Department of Neuroscience, Faculty of Medicine, Mashhad University of Medical Sciences, Mashhad, Iran

^3^ Shefa Neuroscience Research Center, Khatam Alanbia Hospital, Tehran, Iran

*Correspondence: Department of Neuroscience, Faculty of Medicine, Mashhad University of Medical Sciences, Pardis Campus, Azadi Square, Kalantari Blvd, Mashhad, Iran; Tel.: +98-51-38002473; Email: sahabnegahs@mums.ac.ir

**^†^** Negin Ghiyasi Moghaddam and Navidreza Shayan contributed equally to this work as second authors.

**Ethics declarations**

**Conflict of interest**

The authors declare no competing interests; this manuscript has not been published or submitted elsewhere. All authors are in agreement with the content of the manuscript.

| **Supplementary Table 1.**  **PRISMA (Preferred Reporting Items for Systematic review and Meta-Analysis) 2020 Checklist.** | | | |
| --- | --- | --- | --- |
| **Section and Topic** | **Item #** | **Checklist item** | **Location where item is reported (page)** |
| **TITLE** | | |  |
| Title | 1 | Identify the report as a systematic review. | 1 |
| **ABSTRACT** | | |  |
| Abstract | 2 | See the PRISMA 2020 for Abstracts checklist. | 1 |
| **INTRODUCTION** | | |  |
| Rationale | 3 | Describe the rationale for the review in the context of existing knowledge. | 2,3 |
| Objectives | 4 | Provide an explicit statement of the objective(s) or question(s) the review addresses. | 2,3 |
| **METHODS** | | |  |
| Eligibility criteria | 5 | Specify the inclusion and exclusion criteria for the review and how studies were grouped for the syntheses. | 3,4 |
| Information sources | 6 | Specify all databases, registers, websites, organisations, reference lists and other sources searched or consulted to identify studies. Specify the date when each source was last searched or consulted. | 3,4 |
| Search strategy | 7 | Present the full search strategies for all databases, registers and websites, including any filters and limits used. | 3, Table S2 |
| Selection process | 8 | Specify the methods used to decide whether a study met the inclusion criteria of the review, including how many reviewers screened each record and each report retrieved, whether they worked independently, and if applicable, details of automation tools used in the process. | 3,4 |
| Data collection process | 9 | Specify the methods used to collect data from reports, including how many reviewers collected data from each report, whether they worked independently, any processes for obtaining or confirming data from study investigators, and if applicable, details of automation tools used in the process. | 3 |
| Data items | 10a | List and define all outcomes for which data were sought. Specify whether all results that were compatible with each outcome domain in each study were sought (e.g. for all measures, time points, analyses), and if not, the methods used to decide which results to collect. | 3,4 |
|  | 10b | List and define all other variables for which data were sought (e.g. participant and intervention characteristics, funding sources). Describe any assumptions made about any missing or unclear information. | n/a |
| Study risk of bias assessment | 11 | Specify the methods used to assess risk of bias in the included studies, including details of the tool(s) used, how many reviewers assessed each study and whether they worked independently, and if applicable, details of automation tools used in the process. | Table S3 |
| Effect measures | 12 | Specify for each outcome the effect measure(s) (e.g. risk ratio, mean difference) used in the synthesis or presentation of results. | n/a |
| Synthesis methods | 13a | Describe the processes used to decide which studies were eligible for each synthesis (e.g. tabulating the study intervention characteristics and comparing against the planned groups for each synthesis (item #5)). | Figure 1 |
|  | 13b | Describe any methods required to prepare the data for presentation or synthesis, such as handling of missing summary statistics, or data conversions. | n/a |
|  | 13c | Describe any methods used to tabulate or visually display results of individual studies and syntheses. | n/a |
|  | 13d | Describe any methods used to synthesize results and provide a rationale for the choice(s). If meta-analysis was performed, describe the model(s), method(s) to identify the presence and extent of statistical heterogeneity, and software package(s) used. | 4 |
|  | 13e | Describe any methods used to explore possible causes of heterogeneity among study results (e.g. subgroup analysis, meta-regression). | 4 |
|  | 13f | Describe any sensitivity analyses conducted to assess robustness of the synthesized results. | n/a |
| Reporting bias assessment | 14 | Describe any methods used to assess risk of bias due to missing results in a synthesis (arising from reporting biases). | n/a |
| Certainty assessment | 15 | Describe any methods used to assess certainty (or confidence) in the body of evidence for an outcome. | n/a |
| **RESULTS** | | |  |
| Study selection | 16a | Describe the results of the search and selection process, from the number of records identified in the search to the number of studies included in the review, ideally using a flow diagram. | 5,6 |
|  | 16b | Cite studies that might appear to meet the inclusion criteria, but which were excluded, and explain why they were excluded. | 5,6 |
| Study characteristics | 17 | Cite each included study and present its characteristics. | Table 1 |
| Risk of bias in studies | 18 | Present assessments of risk of bias for each included study. | Table S3 |
| Results of individual studies | 19 | For all outcomes, present, for each study: (a) summary statistics for each group (where appropriate) and (b) an effect estimate and its precision (e.g. confidence/credible interval), ideally using structured tables or plots. | All figures |
| Results of syntheses | 20a | For each synthesis, briefly summarise the characteristics and risk of bias among contributing studies. | Figures |
|  | 20b | Present results of all statistical syntheses conducted. If meta-analysis was done, present for each the summary estimate and its precision (e.g. confidence/credible interval) and measures of statistical heterogeneity. If comparing groups, describe the direction of the effect. | Figures |
|  | 20c | Present results of all investigations of possible causes of heterogeneity among study results. | 11 |
|  | 20d | Present results of all sensitivity analyses conducted to assess the robustness of the synthesized results. | n/a |
| Reporting biases | 21 | Present assessments of risk of bias due to missing results (arising from reporting biases) for each synthesis assessed. | Table s3 |
| Certainty of evidence | 22 | Present assessments of certainty (or confidence) in the body of evidence for each outcome assessed. | n/a |
| **DISCUSSION** | | |  |
| Discussion | 23a | Provide a general interpretation of the results in the context of other evidence. | 9,10,11 |
|  | 23b | Discuss any limitations of the evidence included in the review. | 11 |
|  | 23c | Discuss any limitations of the review processes used. | 11 |
|  | 23d | Discuss implications of the results for practice, policy, and future research. | 11,12 |
| **OTHER INFORMATION** | | |  |
| Registration and protocol | 24a | Provide registration information for the review, including register name and registration number, or state that the review was not registered. | n/a |
|  | 24b | Indicate where the review protocol can be accessed, or state that a protocol was not prepared. | n/a |
|  | 24c | Describe and explain any amendments to information provided at registration or in the protocol. | n/a |
| Support | 25 | Describe sources of financial or non-financial support for the review, and the role of the funders or sponsors in the review. | 12,13 |
| Competing interests | 26 | Declare any competing interests of review authors. | 12,13 |
| Availability of data, code and other materials | 27 | Report which of the following are publicly available and where they can be found: template data collection forms; data extracted from included studies; data used for all analyses; analytic code; any other materials used in the review. | n/a |

| Supplementary Table 2. The key search strategy used in the meta-analysis | | |
| --- | --- | --- |
| No. | **Databases** | **Search Terms** |
| 1 | PubMed | ((((((((((((hepatic encephalopathy[Title/Abstract]) OR (hepatic coma[Title/Abstract])) OR (Portal-Systemic Encephalopathy[Title/Abstract])) OR (Hepatocerebral Encephalopathy[Title/Abstract])) OR (Portosystemic Encephalopathy[Title/Abstract])) OR (Fulminant Hepatic Failure with Cerebral Edema[Title/Abstract])) OR (Hepatic Stupor[Title/Abstract])) AND (ammonia[Title/Abstract]))) OR (hyperammonemia[Title/Abstract])) OR (hyperammonemic[Title/Abstract])) OR (NH3) |
| 2 | Scopus | TITLE-ABS-KEY ( hepatic AND encephalopathy ) OR TITLE-ABS-KEY ( hepatic AND coma ) OR TITLE-ABS-KEY ( portal-systemic AND encephalopathy ) OR TITLE-ABS-KEY ( portal AND systemic AND encephalopathy ) OR TITLE-ABS-KEY ( hepatocerebral AND encephalopathy ) OR TITLE-ABS-KEY ( portosystemic AND encephalopathy ) OR TITLE-ABS-KEY ( fulminant AND hepatic AND failure AND with AND cerebral AND edema ) OR TITLE-ABS-KEY ( hepatic AND stupor ) AND TITLE-ABS-KEY ( ammonia ) OR TITLE-ABS-KEY ( hyperammonemia ) OR TITLE-ABS-KEY ( hyperammonemic ) OR TITLE-ABS-KEY ( nh3 ) AND ( LIMIT-TO ( DOCTYPE , "ar" ) ) AND ( LIMIT-TO ( PUBSTAGE , "final" ) ) AND ( LIMIT-TO ( LANGUAGE , "English" ) ) AND LIMIT-TO ( EXACTKEYWORD , "Clinical Article" ) OR LIMIT-TO ( EXACTKEYWORD , "Major Clinical Study" ) ) |
| 3 | Web of Science | (((((((((((((((((AB=(hepatic encephalopathy)) OR AB=(hepatic coma)) OR AB=(Portal-Systemic Encephalopathy)) OR AB=(Portal Systemic Encephalopathy)) OR AB=(Hepatocerebral Encephalopathy)) OR AB=(Portosystemic Encephalopathy)) OR AB=(Fulminant Hepatic Failure with Cerebral Edema)) OR AB=(Hepatic Stupor)) AND AB=(ammonia)) OR AB=(hyperammonemia)) OR AB=(hyperammonemic)) OR AB=(NH3)) AND TS=(Human)) AND |
| 4 | Embase | ('hepatic encephalopathy':ab,ti OR 'hepatic coma':ab,ti OR 'hepatocerebral encephalopathy':ab,ti OR 'portosystemic encephalopathy':ab,ti OR 'fulminant hepatic failure with cerebral edema':ab,ti OR 'hepatic stupor':ab,ti) AND ammonia:ab,ti OR hyperammonemia:ab,ti OR hyperammonemic:ab,ti OR nh3:ab,ti AND ('clinical article'/ de OR 'human'/ de OR 'observational study'/de OR 'pilot study'/ AND 'article'/it |
| 5 | VHL | (hepatic encephalopathy) OR (hepatic coma) OR (portal systemic encephalopathy) OR (hepatocerebral encephalopathy) OR (fulminant hepatic failure with cerebral edema) OR (portosystemic encephalopathy) AND (ammonia) OR (hyperammonemia) OR (hyperammonemic) OR (nh3) AND ( type_of_study:("observational_studies") AND la:("en") AND type:("article")) |

**Supplementary Methods**

**Supplementary Results**

| **Supplementary Table 3.** Risk of bias paper’s judgments for each included study, evaluated by the Joanna Briggs Institute (JBI). | | | | | | | | | | |
| --- | --- | --- | --- | --- | --- | --- | --- | --- | --- | --- |
| **1. Were the groups comparable other than presence of disease in cases or absence of disease in controls?**  **2. Were cases and controls matched appropriately?**  **Author-Year** | **3. Were the same criteria used for identification of cases and controls?** | **4. Was exposure measured in a standard, valid and reliable way?** | **5. Was exposure measured in the same way for cases and controls?** | **6. Were confounding factors identified?** | **7. Were strategies to deal with confounding factors stated?** | **8. Were outcomes assessed in a standard, valid and reliable way for cases and controls?** | **9. Was the exposure period of interest long enough to be meaningful?** | **10. Was appropriate statistical analysis used?** |  |  |
| **Tran et al. 2021** | **+** | **+** | **+** | **+** | **+** | **+** | **+** | **+** | **×** | **+** |
| **Ampuero et al. 2020** | **+** | **+** | **+** | **+** | **+** | **+** | **+** | **+** | **×** | **+** |
| **Tsai et al. 2019** | **+** | **+** | **+** | **+** | **+** | **+** | **+** | **+** | **×** | **+** |
| **Nardelli et al. 2019** | **+** | **+** | **+** | **+** | **+** | **+** | **+** | **+** | **×** | **+** |
| **Zhang et al. 2018** | **+** | **+** | **+** | **+** | **+** | **-** | **-** | **+** | **×** | **+** |
| **Lu et al. 2018** | **+** | **+** | **+** | **+** | **+** | **-** | **-** | **+** | **×** | **+** |
| **Cheng et al. 2018** | **+** | **+** | **+** | **+** | **+** | **-** | **-** | **+** | **×** | **+** |
| **Zheng et al. 2017** | **+** | **+** | **+** | **+** | **+** | **-** | **-** | **+** | **×** | **+** |
| **Zhang et al. 2017** | **+** | **+** | **+** | **+** | **+** | **-** | **-** | **+** | **×** | **+** |
| **Zhou et al. 2016** | **+** | **+** | **+** | **+** | **+** | **-** | **-** | **+** | **×** | **+** |
| **Thomsen et al. 2016** | **+** | **+** | **+** | **+** | **+** | **+** | **+** | **+** | **×** | **+** |
| **Schiff et al. 2016** | **+** | **+** | **+** | **+** | **+** | **-** | **-** | **+** | **×** | **+** |
| **Iwasa et al. 2016** | **+** | **+** | **-** | **+** | **+** | **+** | **+** | **+** | **×** | **+** |
| **Rodríguez et al. 2015** | **+** | **+** | **+** | **+** | **+** | **+** | **+** | **+** | **×** | **+** |
| **Jao et al. 2015** | **+** | **+** | **+** | **+** | **+** | **-** | **-** | **+** | **×** | **+** |
| **Barbosa et al. 2015** | **+** | **+** | **+** | **+** | **+** | **-** | **-** | **+** | **×** | **+** |
| **Zheng et al. 2014** | **+** | **+** | **+** | **+** | **+** | **-** | **-** | **+** | **×** | **+** |
| **Zhang et al. 2014** | **+** | **+** | **+** | **+** | **+** | **-** | **-** | **+** | **×** | **+** |
| **Felipo et al. 2014** | **+** | **+** | **+** | **+** | **+** | **+** | **+** | **+** | **×** | **+** |
| **Zhang et al. 2013** | **+** | **+** | **+** | **+** | **+** | **+** | **+** | **+** | **×** | **+** |
| **Luo et al. 2013** | **+** | **+** | **+** | **+** | **+** | **+** | **+** | **+** | **×** | **+** |
| **Felipo et al. 2013** | **+** | **+** | **+** | **+** | **+** | **+** | **+** | **+** | **×** | **+** |
| **Michalska et al. 2013** | **+** | **+** | **-** | **+** | **+** | **-** | **-** | **+** | **×** | **+** |
| **Ni et al. 2012** | **+** | **+** | **+** | **+** | **+** | **-** | **-** | **+** | **×** | **+** |
| **Luo et al. 2012** | **+** | **+** | **+** | **+** | **+** | **+** | **+** | **+** | **×** | **+** |
| **Srivastava et al. 2011** | **+** | **-** | **+** | **+** | **+** | **-** | **-** | **+** | **×** | **+** |
| **Gad et al. 2011** | **+** | **+** | **+** | **+** | **+** | **+** | **+** | **+** | **×** | **+** |
| **Sharma et al. 2010** | **+** | **+** | **+** | **+** | **+** | **+** | **+** | **+** | **×** | **+** |
| **Goel et al. 2010** | **+** | **+** | **+** | **+** | **+** | **-** | **-** | **+** | **×** | **+** |
| **Montoliu et al. 2009** | **+** | **+** | **+** | **+** | **+** | **+** | **+** | **+** | **×** | **+** |
| **Montoliu et al. 2007** | **+** | **+** | **+** | **+** | **+** | **-** | **-** | **+** | **×** | **+** |
| **Kundra et al. 2005** | **+** | **+** | **-** | **+** | **+** | **-** | **-** | **+** | **×** | **+** |
| **Nicolao et al. 2003** | **+** | 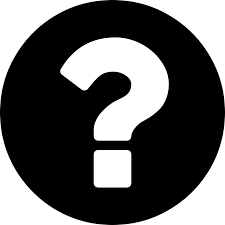 | **+** | **+** | **+** | **-** | **-** | **+** | **×** | **+** |
| **Romero-Go´mez et al. 2001** | **+** | **+** | **+** | **+** | **+** | **+** | **+** | **+** | **×** | **+** |
| **Testa R et al. 1989** | **+** | 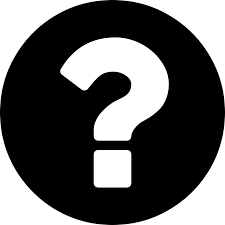 | **+** | **+** | **+** | **-** | **-** | **+** | **×** | **+** |
| **McCLAIN, et al. 1980** | **+** | **+** | 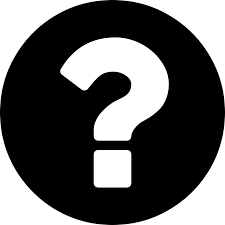 | **+** | **+** | **-** | **-** | **+** | **×** | 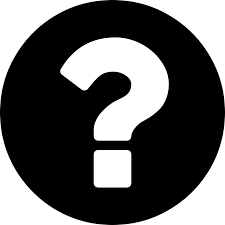 |
| **Reichert et al. 2020** | **+** | **+** | **+** | **+** | **+** | **+** | **+** | **+** | **×** | **+** |
| **Abid et al. 2020** | **+** | **+** | **+** | **+** | **+** | **+** | **+** | **+** | **×** | **+** |
| **Zeng et al. 2019** | **+** | **+** | **+** | **+** | **+** | **+** | **+** | **+** | **×** | **+** |
| **Yousif et al. 2019** | **+** | **+** | **+** | **+** | **+** | **+** | **+** | **+** | **×** | **+** |
| **Yoon et al. 2019** | **+** | **+** | **+** | **+** | **+** | **+** | **+** | **+** | **×** | **+** |
| **Tan et al. 2019** | **+** | **+** | **+** | **+** | **+** | **+** | **+** | **+** | **×** | **+** |
| **Sato et al. 2019** | **+** | **+** | **+** | **+** | **+** | **-** | **-** | **+** | **×** | **+** |
| **Metwally et al. 2019** | **+** | **+** | **+** | **+** | **+** | **-** | **-** | **+** | **×** | **+** |
| **Li et al. 2019** | **+** | **+** | **+** | **+** | **+** | **-** | **-** | **+** | **×** | **+** |
| **Wang et al. 2017** | **+** | **+** | **+** | **+** | **+** | **+** | **+** | **+** | **×** | **+** |
| **Coskun et al. 2017** | **+** | **+** | **+** | **+** | **+** | **-** | **-** | **+** | **×** | **+** |
| **Jeong et al. 2017** | **+** | **+** | **+** | **+** | **+** | **-** | **-** | **+** | **×** | **+** |
| **Ruiz-Margáin et al. 2016** | **+** | **+** | **+** | **+** | **+** | **+** | **+** | **+** | **×** | **+** |
| **Lauridsen et al. 2016** | **+** | **+** | **+** | **+** | **+** | **+** | **+** | **+** | **×** | **+** |
| **Chen et al. 2016** | **+** | **+** | **+** | **+** | **+** | **+** | **+** | **+** | **×** | **+** |
| **Tsai et al. 2015** | **+** | **-** | **+** | **+** | **+** | **+** | **+** | **+** | **×** | **+** |
| **Riggio et al. 2015** | **+** | **+** | **+** | **+** | **+** | **+** | **+** | **+** | **×** | **+** |
| **Wei Li et al. 2015** | **+** | 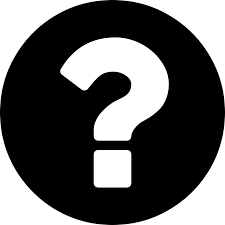 | **+** | **+** | **+** | **+** | **+** | **+** | **×** | **+** |
| **Jindal et al. 2015** | **+** | **+** | **+** | **+** | **+** | **-** | **-** | **+** | **×** | **+** |
| **Patidar et al. 2014** | **+** | **+** | **+** | **+** | **+** | **+** | **+** | **+** | **×** | **+** |
| **Kircheis et al. 2014** | **+** | **+** | **+** | **+** | **+** | **+** | **+** | **+** | **×** | **+** |
| **Hassan et al. 2014** | **+** | **+** | **+** | **+** | **+** | **-** | **-** | **+** | **×** | **+** |
| **Cona et al. 2014** | **+** | **+** | **+** | **+** | **+** | **-** | **-** | **+** | **×** | **+** |
| **Zhang et al. 2013 *** | **+** | **+** | **+** | **+** | **+** | **-** | **-** | **+** | **×** | **+** |
| **Merli et al. 2013** | **+** | **+** | **+** | **+** | **+** | **+** | **+** | **+** | **×** | **+** |
| **Li et al. 2013** | **+** | **+** | **+** | **+** | **+** | **+** | **+** | **+** | **×** | **+** |
| **Sharma et al. 2012** | **+** | **+** | **+** | **+** | **+** | **-** | **-** | **+** | **×** | **+** |
| **Wunsch et al. 2011** | **+** | **+** | **+** | **+** | **+** | **+** | **+** | **+** | **×** | **+** |
| **Riggio et al. 2011** | **+** | **+** | **+** | **+** | **+** | **+** | **+** | **+** | **×** | **+** |
| **Duarte-Rojo et al. 2011** | **+** | **-** | **+** | **+** | **+** | **+** | **+** | **+** | **×** | **+** |
| **Tan et al. 2009** | **+** | **-** | **+** | **+** | **+** | **+** | **+** | **+** | **×** | **+** |
| **Kircheis et al. 2009** | **+** | **+** | **+** | **+** | **+** | **-** | **-** | **+** | **×** | **+** |
| **Sugimoto et al. 2008** | **+** | **+** | **+** | **+** | **+** | **-** | **-** | **+** | **×** | **+** |
| **Chakrabarti et al. 2002** | **+** | **-** | **+** | **+** | **+** | **-** | **-** | **+** | **×** | **+** |
| **Alvarez-Leal et al. 2001** | **+** | **+** | **+** | **+** | **+** | **-** | **-** | **+** | **×** | **+** |
| **Lee et al. 1999** | **+** | **+** | **+** | **+** | **+** | **-** | **-** | **+** | **×** | **+** |
| **Zheng et al. 2013** | **+** | **+** | **+** | **+** | **+** | **-** | **-** | **+** | **×** | **+** |
| **Tao et al. 2013** | **+** | **+** | **+** | **+** | **+** | **+** | **+** | **+** | **×** | **+** |
| **Iversen et al. 2014** | **+** | **+** | **-** | **+** | **+** | **+** | **+** | **+** | **×** | **+** |
| **Kooka et al. 2016** | **+** | **-** | **+** | **+** | **+** | **+** | **+** | **+** | **×** | **+** |
| **Garcia-Garcia et al. 2017** | **+** | **+** | **+** | **+** | **+** | **+** | **+** | **+** | **×** | **+** |
| **Formentin et al. 2019** | **+** | **+** | **+** | **+** | **+** | **+** | **+** | **+** | **×** | **+** |
| **Mangini et al. 2023** | **+** | **-** | **-** | **+** | **+** | **+** | **+** | **+** | **×** | **+** |
| **Kapoor et al. 2023** | **+** | **+** | **+** | **+** | **+** | **+** | **+** | **+** | **×** | **+** |
| **Fiorillo et al. 2023** | **+** | **+** | **+** | **+** | **+** | **+** | **+** | **+** | **×** | **+** |

**Supplementary Results**

Twenty-six studies were included to compare the average Child-Pugh score between groups (1909 participants). Our analysis revealed that, in comparison to the control group, the mean score of these scores was significantly greater in cirrhotic individuals with HE (P < 0.00001; I^2^ = 62%; Supplementary Figure 1a). Likewise, in 46 included papers containing 4558 participants, the MELD score was statistically higher in HE groups (P < 0.00001; I^2^ = 79%; Supplementary Figure 1b).

Twenty-seven publications were considered to compare PT between groups. (1910 participants). The average time for PT was significantly increased in HE patients (P = 0.004; I^2^ = 85%; Supplementary Figure 2a). Moreover, 21 studies were recruited to compare the INR between patients (1578 participants). The average values for INR were statistically increased in HE groups compared to control (P < 0.00001; I^2^ = 55%; Supplementary Figure 2b).

Furthermore, we included 51 valid studies to evaluate the standardized mean difference of total bilirubin between hepatic encephalopathy (HE) and control groups, involving 4709 participants. Our analysis revealed a significant increase in total bilirubin levels in cirrhotic patients with HE compared to those without HE (P < 0.00001; I^2^ = 65%; see Supplementary Figure 3).

To compare the circulating levels of ALT between the case and control groups, we considered twenty-nine studies, involving 2644 participants. The analysis indicated that the mean ALT values were statistically higher in HE patients (P = 0.02; I^2^ = 74%; see Supplementary Figure 4a). Additionally, we used 26 studies to compare the average levels of AST between the control group (N = 1060) and the HE group (N = 678) Our meta-analysis using random effects revealed increased blood levels of AST in HE patients in comparison to control (P = 0.0001; I^2^ = 68%; Supplementary Figure 4b). We also included 8 papers to compare the mean levels of GGT between groups (322 participants). The findings demonstrated that increased circulating levels of GGT in HE patients in comparison to cirrhotic-control (P = 0.006; I^2^ = 1%; Supplementary Figure 4c).

In addition, 12 papers with 1130 patients were recruited to compare the hemoglobin levels of the HE and control groups. Our findings showed that there was no difference in the average hemoglobin levels across the groups (P = 0.09; I^2^ = 78%; Supplementary Figure 5a). To compare the number of platelets in each group, eighteen publications with 2084 participants were considered. The results showed a significant decreased in the number of platelets in HE patients in comparison to cirrhotic-controls (P = 0.08; I^2^ = 67%; Supplementary Figure 5b). We also included 14 individual studies to compare the average number of circulatory white blood cells between HE and control groups (totally 1313 participants). The number of circulatory white blood cells was not statistically different between both groups (P = 0.28; I^2^ = 49%; Supplementary Figure 5c).

**Supplementary Figure 1.** Forest plot for estimating the standardized mean difference of Child-Pugh (a), and MELD scores (b) between control and HE groups.

**Supplementary Figure 2.** Forest plot for estimating the standardized mean difference of circulatory PT (a) and values of INR (b) between control and HE groups.

**Supplementary Figure 3.** Forest plot for estimating the standardized mean difference of circulatory total bilirubin between control and HE groups.

**Supplementary Figure 4.** Forest plot for estimating the standardized mean difference of circulatory ALT (a), AST (b), and GGT (c) between control and HE groups.

**Supplementary Figure 5.** Forest plot for estimating the standardized mean difference of hemoglobin (a), platelets (b) and white blood cells (c) between control and HE groups.
