# Supplementary figures and images for: Correlation of ammonia and blood laboratory parameters with hepatic encephalopathy: A systematic review and meta-analysis
Source: PLoS One. 2024 Sep 3;19(9):e0307899. doi: 10.1371/journal.pone.0307899 (PMC11371226; doi:10.1371/journal.pone.0307899)

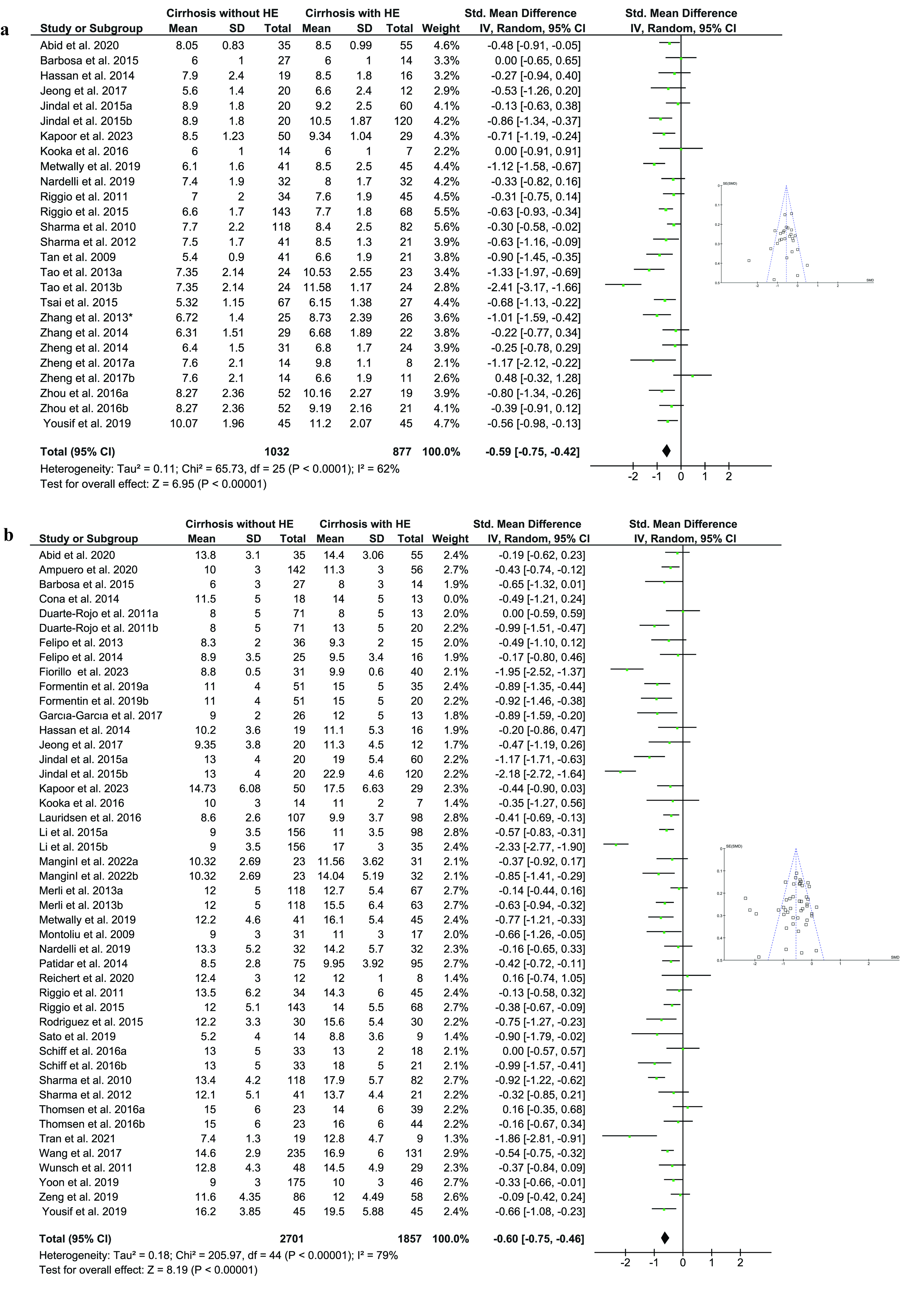

Supplement: S1 Fig — (TIF) [file pone.0307899.s002.tif]

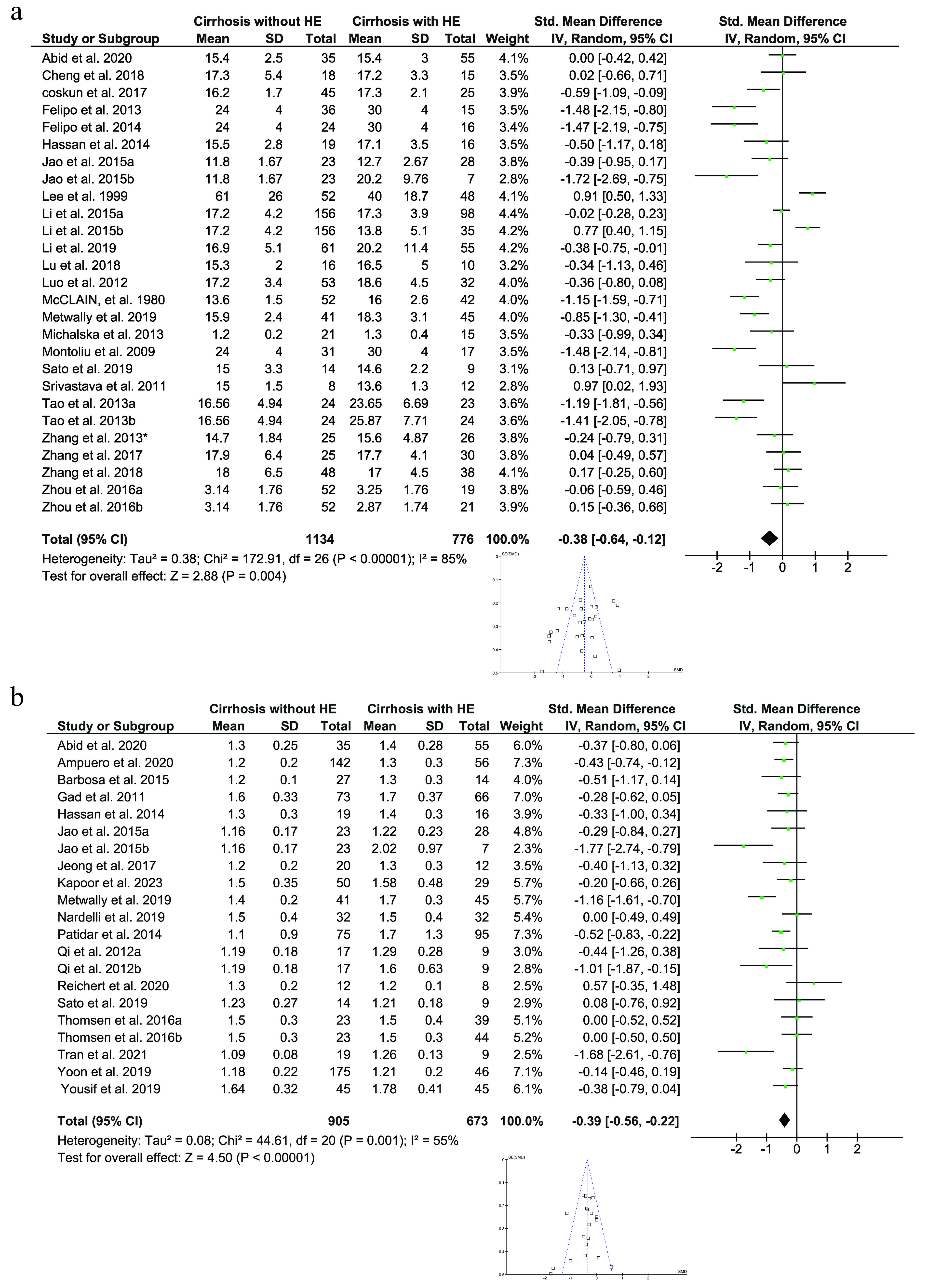

Supplement: S2 Fig — (TIF) [file pone.0307899.s003.tif]

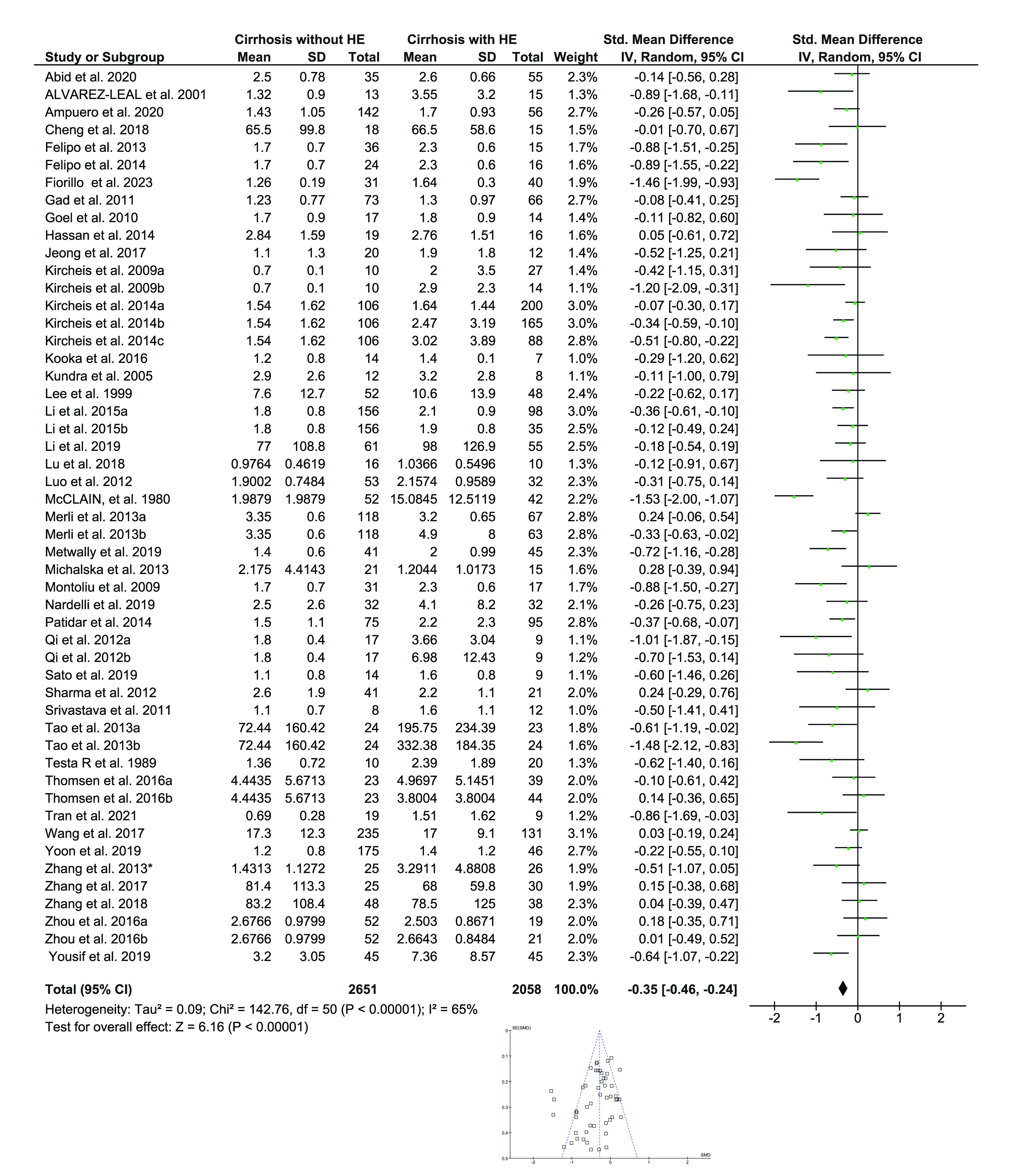

Supplement: S3 Fig — (TIF) [file pone.0307899.s004.tif]

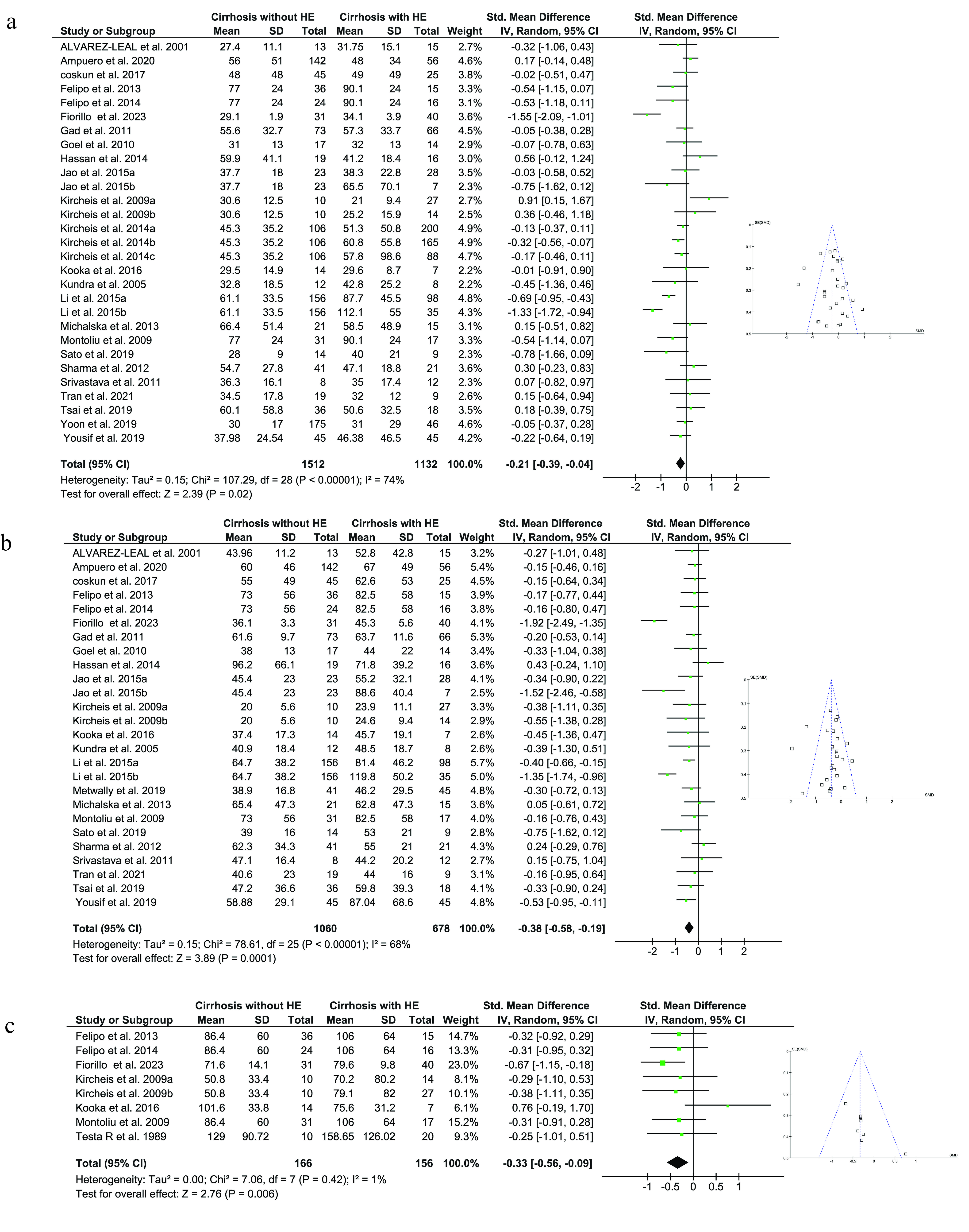

Supplement: S4 Fig — (TIF) [file pone.0307899.s005.tif]

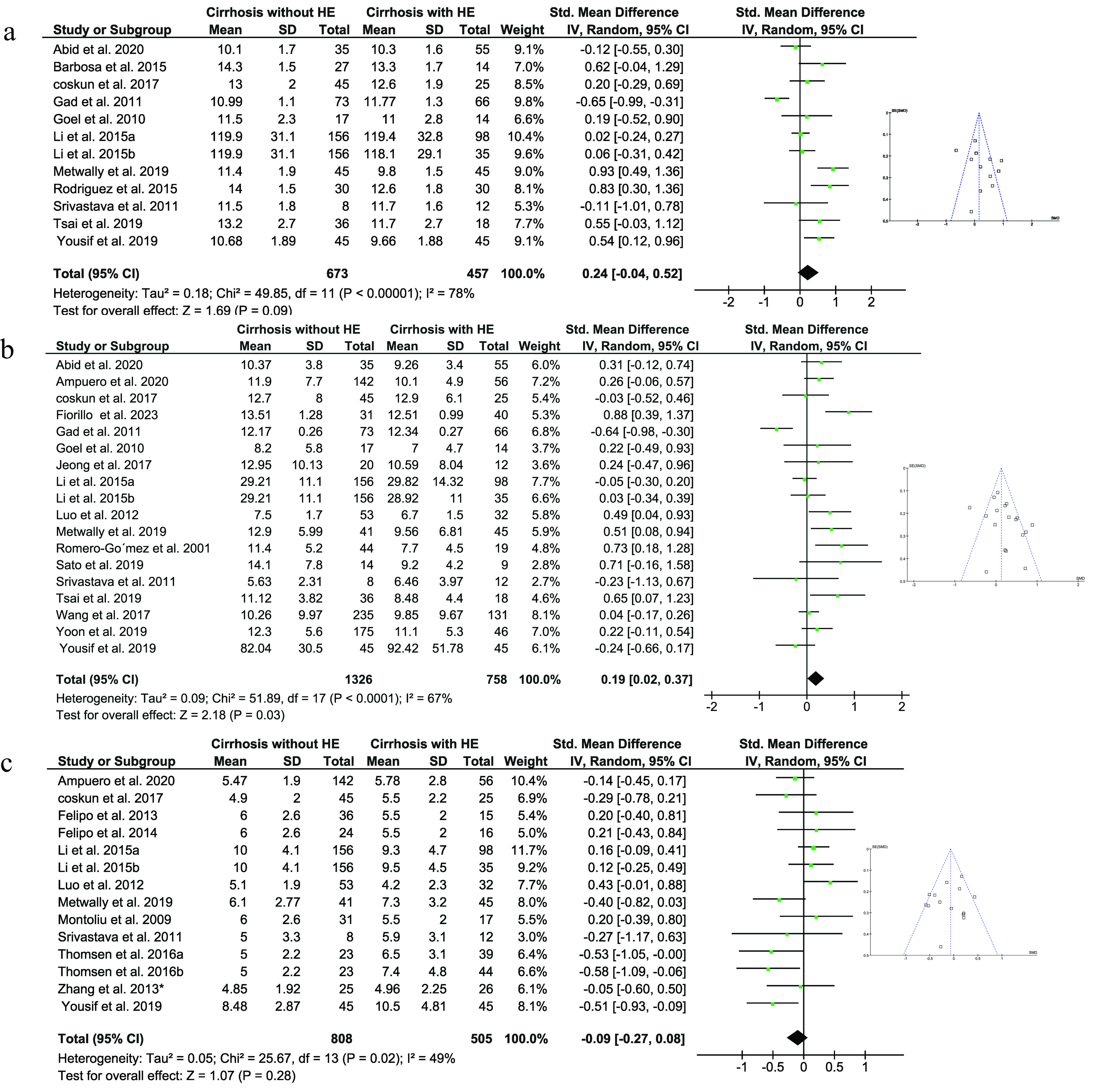

Supplement: S5 Fig — (TIF) [file pone.0307899.s006.tif]
